# IgE Epitopes of the House Dust Mite Allergen Der p 7 Are Mainly Discontinuous and Conformational

**DOI:** 10.3389/fimmu.2021.687294

**Published:** 2021-06-15

**Authors:** Mirela Curin, Huey-Jy Huang, Tetiana Garmatiuk, Sandra Gutfreund, Yvonne Resch-Marat, Kuan-Wei Chen, Kerstin Fauland, Walter Keller, Petra Zieglmayer, René Zieglmayer, Patrick Lemell, Friedrich Horak, Wolfgang Hemmer, Margarete Focke-Tejkl, Sabine Flicker, Susanne Vrtala, Rudolf Valenta

**Affiliations:** ^1^ Division of Immunopathology, Department of Pathophysiology and Allergy Research, Center for Pathophysiology, Infectiology and Immunology, Medical University of Vienna, Vienna, Austria; ^2^ Institute of Molecular Biosciences, University of Graz, Graz, Austria; ^3^ Vienna Challenge Chamber, Vienna, Austria; ^4^ Karl Landsteiner University of Health Sciences, Krems, Austria; ^5^ Allergy Center Vienna, West, Vienna, Austria; ^6^ FAZ-Floridsdorf Allergy Center, Vienna, Austria; ^7^ Department of Clinical Immunology and Allergy, Sechenov First State Medical University, Moscow, Russia; ^8^ NRC Institute of Immunology FMBA of Russia, Moscow, Russia

**Keywords:** allergy, allergen, allergen structure, house dust mite allergy, Der p 7, IgE epitope mapping, peptides

## Abstract

**Background:**

Several studies indicate that Der p 7 is an important and clinically relevant allergen of *Dermatophagoides pteronyssinus* which should be included in vaccines for treatment of house dust mite (HDM) allergy. Aim of this study was to characterize the IgE epitopes of Der p 7.

**Methods:**

Recombinant Der p 7 was expressed and purified, analyzed for fold by circular dichroism and tested for its allergenic activity by basophil activation. Seven overlapping, surface-exposed peptides (P1–P7) with a length of 27 to 37 amino acids, which spanned the Der p 7 sequence, were synthesized and tested for IgE reactivity and allergenic activity by basophil activation assay. Carrier-bound peptides were studied for their ability to induce allergen-specific IgG antibodies in rabbits. Peptide-specific antibodies were used to inhibit allergic patients` IgE binding to Der p 7 by ELISA for mapping of IgE epitopes.

**Results:**

rDer p 7 showed high allergenic activity comparable with Der p 5, Der p 21, and Der p 23. None of the seven tested peptides showed any IgE reactivity or allergenic activity when tested with HDM- allergic patients indicating lack of sequential IgE epitopes on Der p 7. IgE inhibition experiments using anti-peptide specific IgGs and molecular modeling enabled us to identify discontinuous, conformational IgE epitopes of Der p 7.

**Conclusion and Clinical Relevance:**

IgE epitopes of Der p 7 belong to the conformational and discontinuous type whereas sequential Der p 7 peptides lack IgE reactivity. It should thus be possible to construct hypoallergenic vaccines for Der p 7 based on carrier-bound allergen peptides.

## Introduction

House dust mites (HDM) are one of the most relevant allergen sources worldwide (1). Contact to HDM is perennial and depending on the region, prevalence of sensitization to HDM is usually higher than 40% within atopic populations (2). Mites are present in the indoor environments, and it is impossible to eliminate them completely from the human habitats. HDM sensitized patients suffer from a range of allergic symptoms, especially from respiratory symptoms, such as asthma and rhinitis as well as from skin allergy (1). HDM is a complex allergen source with more than 20 different reported allergen groups but only certain allergens are of high clinical relevance (3). These include the major allergens, Der p 1, Der p 2, and Der p 23 which have a prevalence of IgE recognition >60% and a group of mid-tier allergens which in some populations are recognized by 50% of HDM allergic patients (4).

In this study we focused on Der p 7 which according to recent studies seems to be a very important HDM allergen. Originally, Der p 7 was categorized as mid-tier group of mite allergens with a frequency of IgE binding 17% to 52% depending on the tested population (4–8). A very recent study reported an IgE prevalence of 56% for Der p 7 in a South African population, and accordingly, Der p 7 was the second most common sensitizing allergen after Der p 23 in this population (9). The clinical relevance of Der p 7 was emphasized already in the study by Lynch et al. who reported that Der p 7 induced immediate hypersensitivity skin-test reactions in 52% of patients who were positive to HDM extract implicating its strong allergenic activity and Der p 7 accounted for binding of one fifth of HDM-specific IgE levels (10). A study performed in clinically well-defined HDM-allergic children showed that Der p 7 was significantly more often recognized by asthmatic than by non-asthmatic children (11). Another study comparing asymptomatic HDM-sensitized and HDM-allergic rhinitis patients showed that symptomatic patients were more frequently sensitized to Der p 7 (12). All these data indicating that Der p 7 is a clinically important HDM allergen which should be included in vaccines for allergen-specific immunotherapy (AIT) of HDM allergy got further support by studies analyzing patients who were treated by HDM-specific AIT.

Allergen-specific immunotherapy (AIT) is the only allergen-specific form of treatment with disease-modifying and long-lasting effects (13–15). However, HDM-specific immunotherapy is only partially successful, and it has been suggested that the absence of important allergens in natural allergen extracts used for vaccination could be a reason for incomplete treatment success (16). This assumption was confirmed by two recent studies demonstrating that AIT with allergen extracts lacking certain HDM allergens such as Der p 7 was less effective in patients sensitized to the missing allergens as compared to patients who were mainly sensitized to Der p 1 and Der p 2 which were included in the vaccines (17, 18). Molecular allergy vaccines allow to include all relevant allergen molecules of a given allergen source but the detailed knowledge of IgE- and T cell epitopes of each of the allergen molecules is crucial for their development (19–21).

The crystal structure of Der p 7 has been solved but little information is available regarding the binding of patient`s IgE to Der p 7 (22). In this study, we therefore investigated the IgE binding epitopes of Der p 7. Seven overlapping peptides of 27 to 37 amino acids with good surface exposure that cover the Der p 7 sequence, were synthesized, and peptide-specific antibodies were raised in rabbits. Using the synthetic peptides and HDM allergic patients’ sera, the presence of sequential/linear IgE epitopes in Der p 7 was investigated by immunoblot and basophil activation assay. Additionally, peptide-specific antibodies were used for competing with the HDM allergic patients’ IgE binding to Der p 7 to search for conformational/discontinuous IgE epitopes. These two approaches enabled us to identify the IgE binding sites recognized by HDM-allergic patients as mainly conformational and discontinuous epitopes on the surface of a 3-dimensional model of the Der p 7 structure.

## Materials And Methods

### Sera From HDM Allergic Patients

To study the clinical relevance of Der p 7, sera from 100 clinically-well characterized HDM-allergic patients had been tested for IgE reactivity to a comprehensive panel of HDM allergen molecules including Der p 7 by ImmunoCAP ISAC technology as described (17). Detailed clinical history of HDM-related respiratory and skin symptoms was available for these patients allowing to analyze associations of Der p 7 IgE positivity with certain clinical symptoms. Since this was a population that was initially selected for AIT, only patients with mild to moderate asthma but not severe asthma were included (17). Additional sera from HDM-allergic patients (n = 37) were obtained from an allergy outpatient clinic in Vienna. These patients had a case history indicative of HDM allergy and a positive skin test result to HDM allergen extract and/or HDM-specific IgE antibodies as measured by house dust mite extract (d1) ImmunoCAP (Thermofisher, Phadia, Uppsala, Sweden) (>0.35 kUA/L). The presence of IgE antibodies to rDer p 7 was determined by dot-blot, ELISA, and/or ImmunoCAP ISAC technology (4, 7, 23).

Serum samples from non-allergic subjects were used as negative controls. All sera samples were analyzed in an anonymous manner after the study with approval of the ethics committee of the Medical University of Vienna, Austria (EK 641/2014). All experiments were performed in accordance with guidelines for good scientific practice of the Medical University of Vienna (https://www.meduniwien.ac.at/web/rechtliches/good-scientific-practice/).

### Synthesis and Coupling of Der p 7-Derived Peptides, Expression, Purification of Folded Recombinant Der p 7, Molecular Modeling

Seven overlapping peptides with a length between 27 and 37 amino acids were identified based on the prediction of surface exposure of amino acids as determined by the ProtScale bioinformatics tool from the ExPASY server (http://web.expasy.org/protscale/) (24). Cysteine residues were added to the N-termini of each of the peptides for the purpose of coupling to KLH. Peptides were synthesized using an Applied Biosystems peptide synthesizer Model 433A (Foster City, CA, USA) and purified by High-performance liquid chromatography (HPLC) (Dionex, Thermofischer Scientific, Waltham, MA, USA) (25). The identity of the synthetic peptides was confirmed by MALDI-TOF analysis (data not shown). For the immunization of rabbits, each of the Der p 7-derived peptides was coupled to keyhole limpet hemocyanin (KLH) (MW 4.5 × 10^5^–1.3 × 10^7^ Daltons; Pierce, ThermoFisher Scientific, Waltham, MA, USA) and purified using a conjugation kit according to manufacturer’s instructions (Pierce, ThermoFisher Scientific). **Table S1** summarizes the position, length, and biochemical properties of the Der p 7-derived synthetic peptides. To produce recombinant folded Der p 7, the sequence coding for Der p 7 optimized for expression in *E. coli* was cloned into pET-17b (Novagen, Maddison, USA), and expression was performed in *E. coli* BL21 (DE3) (Agilent Technologies, Santa Clara, USA) by adding 0.5 mM IPTG to the bacterial cultures and incubation in a GFL 3033 incubator (GFL, Germany) for further 4 h at 37°C. Bacterial pellets were collected by centrifugation for 15 min, at 3,000*g* and frozen at −20°C. Frozen pellets were re-suspended in 25 mM Imidazole, pH 7.4, 0.1% Triton X-100, lysozyme (500 µg) was added, and cells were stirred for 20 min at RT. Three cycles of freezing and thawing (in liquid nitrogen and 50° water bath) were applied to open the cells, followed by addition of 1 µl DNAse (stock: 1 mg/ml) and stirring for 10 min at RT. NaCl was added to a final concentration of 100 mM, and debris was removed by centrifugation at 19,900*g* for 20 min at 4°C. Proteins other than Der p 7 were precipitated from the supernatant by increasing the concentration of ammonium sulfate (0 - 40%: 226 g/L) and stirring the mixture at 4°C for 2 h. Precipitated proteins were removed by centrifugation (30 min, 19,900*g*, 4°C), and additional amount of ammonium sulfate was added (40–60%: 120 g/L) to the supernatant while stirring the mixture at 4°C for 2 h. Precipitated proteins were removed by centrifugation for 30 min, 19,900*g*, at 4°C. Der p 7 accumulated in the supernatant and was further polished by hydrophobic interaction chromatography using a HiTrap™ Phenyl FF column, 5 ml, (GE Healthcare) with solutions A (2 M ammonium sulfate, 50 mM Na_2_HPO_4_, pH 7, 10 mg/L PMSF), and elution solution B (50 mM Na_2_HPO_4_, pH 7, 10 mg/L PMSF). rDer p 7-containing fractions were combined and dialyzed against solution C (10 mM Na_2_HPO_4_, pH 7, 0.25 mM DTT, 0.01 mM NaN_3_, 10 mg/L PMSF) to continue purification by hydroxypatite chromatography using a Bio-Gel HT Hydroxyapatite 5 ml column (BioRad, Hercules, CA, USA). Solution D (300 mM Na_2_HPO_4_, pH 7, 0.25 mM DTT, 0.01 mM NaN_3_, 10 mg/L PMSF) was used for elution of Der p 7. Fractions containing Der p 7 were combined, dialyzed against 10 mM Na_2_HPO_4_, pH 7, and stored at −20°C. Purity and migration pattern of Der p 7 were analyzed by SDS-PAGE.

The secondary structure of rDer p 7 was studied by circular dichroism (CD) spectroscopy using a Jasco J-810 spectropolarimeter (Japan Spectroscopic Co., Tokyo, Japan). Measurements were performed between 250 and 190 nm with a resolution of 0.5 nm at a scanning speed of 50 nm/min. Final spectra were normalized by subtracting the buffer spectrum. Results are shown as mean residue ellipticities at a given wave length. The melting point was determined as described (26). The secondary structure estimation program CDSSTR from the DichroWeb server was used to calculate the secondary structure.

Recombinant Der p 5, Der p 21, and Der p 23 were purified as described (27–29).

A model of Der p 7 was created based on the crystal structure of Der p 7 deposited in the protein data bank (http://www.rcsb.org/pdb/home/home.do; PDB: 3H4Z) (22). The solvent-accessible surface (SAS) of each amino acid residue was calculated with the program MSMS (30). SAS is determined by a spherical solvent probe (r = 1.4 Å) rolling over the van der Waals surface of the protein and is given in Å2. The surface exposure of the peptides was calculated as follows: Surface exposure [%] = SAS of all amino acids of the peptide/SAS of all amino acids of the model*100. Graphic depictions of the model were rendered with PyMOL (PyMOL Molecular Graphics System, Version 1.8.0; Schrödinger, NY).

### IgE Reactivity of Dot-Blotted rDer p 7 and Der p 7-Derived Peptides

Aliquots containing 0.5 µg of purified rDer p 7, each of the Der p 7-derived peptides and for control purposes BSA were dotted onto a nitrocellulose membrane (Schleicher & Schuell, Dassel, Germany). Membranes were blocked with gold buffer (50 mM sodium phosphate [pH 7.4], 0.5% [v/v] Tween-20, 0.5% [w/v] BSA, and 0.05% [w/v] sodium azide), three times for 20 min and then incubated with HDM-allergic patients’ sera (diluted 1:10 in gold buffer) and with a serum from a non-allergic person (1:10 in gold buffer) overnight at 4°C. Bound IgE was detected with 1:10 diluted ^125^I-labeled anti-human IgE Abs (Demeditec Diagnostics, Kiel, Germany) and visualized by autoradiography (Kodak XOMAT film).

### Rat Basophil Leukemia (RBL) Assay for Testing Allergenic Activity

To test the allergenic activity of rDer p 7 and the Der p 7-derived peptides, rat basophil leukemia cells (RBL) expressing human high-affinity IgE receptor FcϵRI (1.5 × 10^5^/well) were loaded overnight with sera from the house dust mite allergic patients and, for control purposes, with the serum from one non-allergic individual at a dilution of 1:20 as described (18). Cells were washed three times with Tyrode’s buffer (Sigma, Vienna, Austria) and exposed to serial dilutions of allergen (43, 4.3, 0.43, and 0.043 nM of rDer p 7, a mix of P1-P7, and a mix of KLH-coupled P1- P7, respectively) for 1 h.

43 nM corresponds to 1,000 ng/ml, 4.3 nM to 100 ng/ml, 0.43 nM to 10 ng/ml and 0.043 nM to 1 ng/ml of rDer p 7.

Cells incubated only with serum but without allergen (marked as serum), and cells plus allergen without serum (marked as buffer) were used to determine the cut-off. Supernatants were analyzed for β-hexosaminidase activity as described previously (31). Experiments were performed in triplicates, and results are presented as mean percentages ± SDs of total β-hexosaminidase released after addition of 1%Triton X-100. To compare the allergenic activity of Der p 7 with Der p 5, Der p 21, and Der p 23, patients’ sera were used in a dilution of 1:20 and all allergens in concentration 100, 10, and 1 ng/ml. Assays were performed as described above. Der p 23 and Der p 5, as well as Der p 21, were used as highly allergenic major and mid-tier allergens, respectively.

### Immunization of Rabbits, Determination of IgG Antibody Levels

Peptide-specific IgG antibodies were obtained by immunizing rabbits three times (first booster injection after 4 weeks and a second booster injection after 7 weeks) with each of the KLH-conjugated peptides (200 µg/injection) and, for control purposes, with recombinant Der p 7 protein (200 µg/injection) (Charles River, Chatillon sur Chalaronnne, France). In total, four rabbits were immunized per peptide/protein, two using Freund’s adjuvant (1× complete and 2× incomplete Freund’s adjuvant) and two rabbits using aluminium hydroxide as adjuvant (SERVA Electrophoresis, Heidelberg, Germany). Pre-immune sera were obtained from the rabbits before immunization. Rabbit immune responses were analyzed by ELISA. For the measurement of specific rabbit IgG antibodies, ELISA plates (Nunc, Roskilde, Denmark) were coated overnight at 4°C with 1 µg/ml of rDer p 7. After blocking, the plates were incubated overnight with serial dilutions of the corresponding rabbit antisera (1:2.000, 1:10.000, and 1:50.000), or the corresponding pre-immune sera (1: 2.000). Bound rabbit IgG antibodies were detected with a 1:2.500 diluted horseradish peroxidase-labeled donkey anti-rabbit IgG antiserum (Amersham Biosciences, Little Chalfont, UK). The optical density (OD) values corresponding to bound antibodies were measured at 405 and 492 nm. All determinations were conducted as duplicates, and results were expressed as mean values with deviations of less than 10%.

### ELISA Competition Assay for Analyzing the Inhibition of Human IgE Binding to rDer p 7 With Peptide-Specific Rabbit Antibodies

ELISA plates were coated overnight with 1 µg/ml rDer p 7 at 4°C, blocked for 2.5 h at 37°C and were then pre-incubated for 24 h with anti-Der p 7 peptide antisera, anti-Der p 7 antisera, or, for control purposes, with the corresponding pre-immune sera at 4°C. Since each peptide was used for immunization of two rabbits with CFA and two rabbits with Al(OH)_3_, anti-sera from two rabbits immunized with the same adjuvant were combined in ratio 1:1 to obtain a total sera dilution of 1:50 for both, CFA-immunized rabbits and Al(OH)_3_-immunized rabbits, respectively. Plates were then washed, and after overnight incubation with sera from HDM-allergic patients (dilution between 1:5 and 1:10), bound IgE antibodies were detected with horseradish peroxidase-labeled goat anti-human IgE antibodies (KPL). The percentage reduction in IgE binding achieved by pre-incubation with rabbit antisera was calculated as follows: 100 − (OD_I/_OD_P_) × 100) where OD_I_ and OD_P_ represent optical density values after pre-incubation with the rabbit immune serum or pre-immune serum, respectively (32, 33).

## Results

### Der p 7 Is an Important HDM Allergen

**Figure 1:**
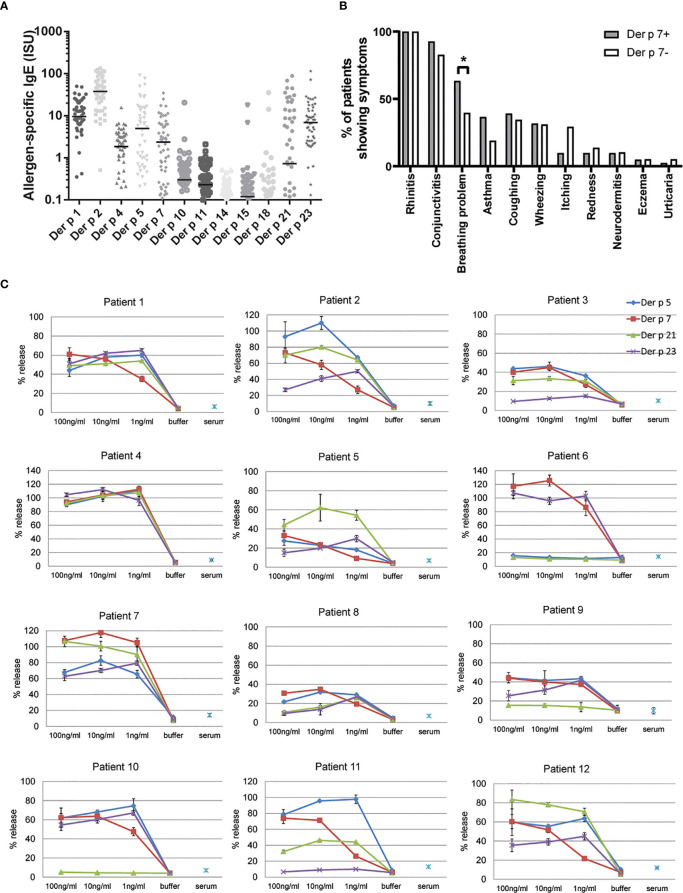
Allergenic activity and possible clinical relevance of Der p 7 **(A)** IgE levels to individual HDM allergens. Specific IgE levels (ISU, y-axis) to HDM allergens(x-axis) determined for patients with clinically confirmed HDM allergy (n = 100) are displayed as scatter plots and represent the means. Median values are depicted as horizontal lines. **(B)** Frequency of allergic symptoms in HDM allergic patients with or without sensitization to Der p 7. Statistically significant differences between the groups are indicated. *P < 0.05. **(C)**. Allergenic activity of Der p 7 in comparison with Der p 5, Der p 21, and Der p 23 as determined by basophil activation testing. RBL cells transfected with human FcϵRI were loaded with sera from house dust mite-allergic patients (1–12) and were then exposed to decreasing concentrations (100, 10, and 1 ng/ml) of Der p 7, Der p 5, Der p 21, and Der p 23. Cells incubated with buffer and allergens (buffer) or with serum without allergens (serum) were used as negative controls. β-hexosaminidase releases are displayed as percentages of total β-hexosaminidase on the y-axes and represent the means of triplicates ± SD.

In this study we used sera from clinically well characterized HDM allergic patients (17) to compare Der p 7-specific IgE levels with IgE levels specific for other HDM allergens and search for associations of Der p 7-specific IgE recognition with clinical phenotypes. **Figure 1** compared Der p 7-IgE levels with IgE levels against other HDM allergens. The levels of allergen-specific IgE antibodies were highest for Der p 2 (median, 37.82; cumulative sum, 2588 ISU), followed by Der p 1 (median, 9.57; sum, 708.8 ISU), Der p 23 (median, 6.9; sum, 638.6 ISU), Der p 5 (median, 4.97; sum, 630.3 ISU), Der p 7 (median, 2.37; sum, 253.8 ISU), Der p 4 (median, 1.85; sum, 127.6 ISU), and Der p 21 (median, 0.73; sum, 638.6 ISU) (**Figure 1**). IgE levels against Der p 10, Der p 11, Der p 14, Der p 15, and Der p 18 were much lower suggesting that these allergens might be less relevant (**Figure 1**). The analysis regarding HDM-related allergic symptoms revealed that patients who had IgE antibodies to Der p 7 more often reported breathing problems and showed higher tendency to suffer from mild asthma than the patients that were not sensitized to Der p 7 (**Figure 1**). In this context, it should be mentioned that only patients with mild to moderate asthma were included in the studied cohort of patients (17).

To study the allergenic activity of Der p 7 we investigated its ability to activate basophils by testing a series of allergen concentrations and sera from HDM allergic patients. We found that Der p 7 could activate basophils at similar low concentrations (i.e., 1–10 ng/ml) like Der p 5, Der p 21, and Der p 23 which have been identified as clinically relevant allergens molecules (**Figure 1**, patients 1–12). Together, these data indicated that Der p 7 is an important and highly potent allergen and should therefore be included in a HDM vaccine.

### Characterization of Overlapping Synthetic Peptides Derived From Solvent Accessible Areas of Der p 7

**Figure 2:**
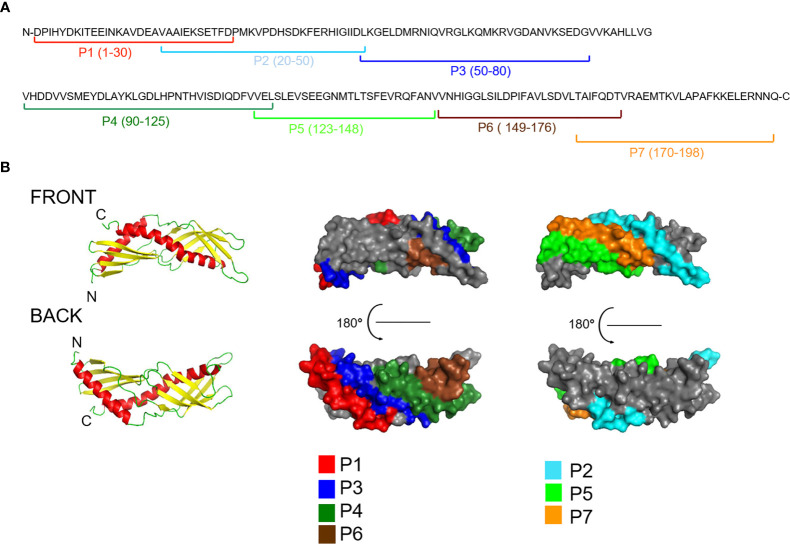
Visualization of peptides spanning the Der p 7 sequence in the Der p 7 structure **(A)** The position of the peptides is indicated in the Der p 7 amino acid sequence (P1-7). **(B)** Visualization of the peptides in a model of the 3-dimensional structure of Der p 7 created according to the crystal structure (PDB: 3H4Z). Left images: Ribbon representations of the Der p 7 structure from the front and back. Central and right images: corresponding surface representations of the Der p 7 structure with the peptides highlighted in different colors (P1, red; P2, turquoise; P3, blue; P4, dark green; P5, green; P6, brown; P7, orange).

Solvent accessibility prediction revealed that the regions of Der p 7 with predicted high surface accessibility can be covered with seven peptides (P1-P7), having a length from 27 to 37 amino acids (**Table S1**; **Figures 2**, **S1**). The coverage of surface by the individual Der p 7 peptides was as follows: peptide 1: 18.2%, peptide 2: 19%, peptide 3: 15.7%, peptide 4: 15.7%, peptide 5: 12.7%, peptide 6: 13% and peptide 7: 13% (**Figure S1**).

**Figure 2** shows a ribbon (left) and surface representation (right) of the Der p 7 molecule. The N-terminal and C-terminal ends are indicated in the ribbon representations, and peptides are colored in the surface representations. In a given view of the Der p 7-structure P2, P5, and P7 appear on the front side, whereas P1, P3, P4, and P6 are located on the backside of the structure (**Figure 2**). Peptides P2, P5, and P7 appear in close vicinity on the front side of the molecule although they are not adjacent in the Der p 7 sequence. Likewise, P1 and P6 appear in the vicinity of the area defined by peptides 3 and 4 on the backside although they are not adjacent to these peptides in the sequence.

### Der p 7-Derived Peptides Lack IgE Reactivity and Allergenic Activity

**Figure 3:**
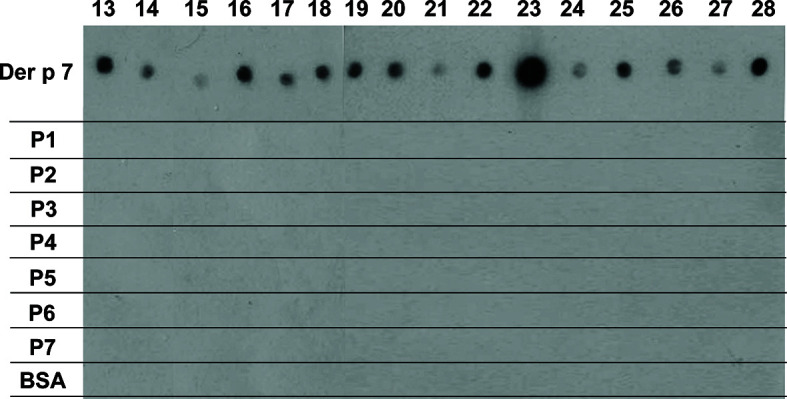
Der p 7 peptides lack IgE reactivity. Dot-blotted Der p 7, Der p 7-derived peptides (P1-P7) and BSA as a negative control were tested for IgE reactivity with sera from 16 HDM-allergic patients (patients 19, 20, 21, 22, 24–28; **Table 1**). Bound IgE antibodies were detected with ^125^I-labeled anti-human IgE antibodies and visualized by autoradiography.

Recombinant Der p 7 could be expressed and purified as a folded and monomeric protein. **Figure S2** showed that rDer p 7 was pure and migrated as a distinct band at approximately 22 kDa in SDS-PAGE. The circular dichroism spectrum of Der p 7 was compatible with a protein assuming a mixed alpha helical-beta sheet fold with a melting point of approximately 60°C (**Figure S2B**). The IgE-binding capacity of folded rDer p 7 was compared with that of the seven Der p 7-derived peptides in a non-denaturing RAST-based IgE-binding dot-blot assay using sera from 16 HDM-allergic patients (**Figure 3**, lanes 13–28). Each of the patients showed IgE reactivity to rDer p 7, and none of the patients showed reactivity to BSA (**Figure 3**). Regarding Der p 7-derived peptides, complete lack of IgE reactivity was found for each of the seven peptides indicating that allergic patients primarily recognize conformational IgE epitopes on Der p 7 and that sequential epitopes play no relevant role for IgE recognition of Der p 7 (**Figure 3**).

**Figure 4:**
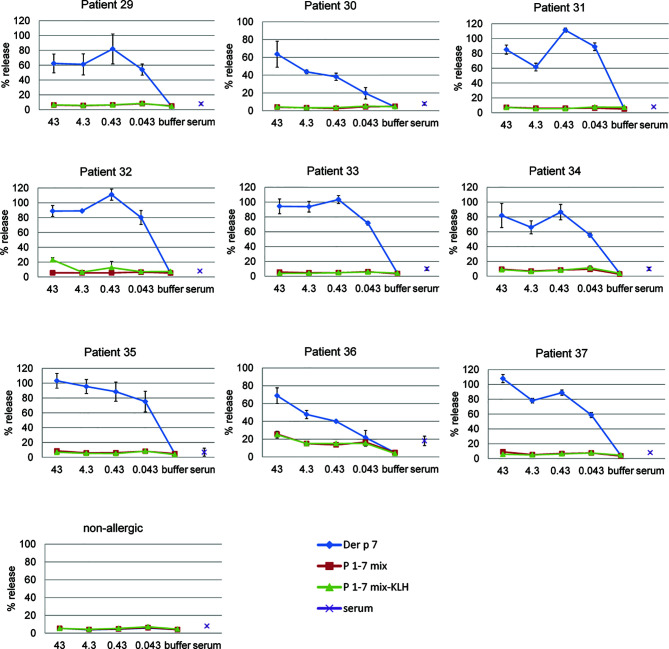
Allergenic activity of rDer p 7 and Der p 7-derived peptides as determined by basophil activation. RBL cells transfected with human FcϵRI were loaded with serum IgE of HDM-allergic patients (patients 29–37) or with serum IgE from a non-allergic person and were then exposed to decreasing concentrations (43, 4.3, 0.43, and 0.043 nM) of rDer p 7, or equimolar concentrations of the peptide mix (P1-7 mix) or a mix of KLH coupled peptides (P1-7 mix-KLH). β-hexosaminidase releases are displayed as percentages of total β-hexosaminidase on the y-axes and represent the mean of triplicates± SD.

Next, we investigated by basophil activation if Der p 7-derived peptides may exhibit some allergenic activity, in particular when they are mixed (**Figure 4**). Additionally, we investigated if coupling of several peptides to a carrier protein KLH may render the peptides allergenic. We found that Der p 7 induced a dose-dependent release of β-hexosaminidase from basophils loaded with serum IgE from HDM-allergic patients (patients 29–37) already at 0.043 nM whereas the equimolar mix of P1, P2, P3, P4, P5, P6, and P7 and an equimolar mix of the KLH-coupled peptides did not activate the basophils up to 43 nM (**Figure 4**). When basophils were loaded with serum IgE from a non-allergic person (NA), no release was observed upon incubation with Der p 7 or Der p 7-derived peptides (**Figure 4**). Incubation of cells with serum alone did not induce basophil degranulation (**Figure 4**).

### Immunization With KLH-Coupled Der p 7-Derived Peptides Induces IgG Antibodies Recognizing Folded Der p 7

Immunization of rabbits with each of the seven KLH-coupled peptides (P1-P7) induced IgG antibodies that recognized folded rDer p 7 at least up to a dilution of 1:2.000 (**Figure S3**). However, the levels of Der p 7-specific IgG antibodies induced by peptide immunization varied depending on the peptide used and in most cases were higher when Freund’s adjuvant was used as compared to aluminium hydroxide (**Figure S3**). The highest levels of Der p 7-specific IgG antibodies were induced by immunization with folded rDer p 7 but the levels induced by P1 were almost comparable (**Figure S3**). Peptides P2, P3, P4, and P7 showed a lower but comparable good immunogenicity yielding Der p 7-specific titers of 1:10.000–1:50.000 (**Figure S3**). Peptides P5 and P6 induced the lowest Der p 7-specific IgG responses which were detectable only at a dilution of 1:2000 (**Figure S3**).

### Localization of IgE Epitopes on the Folded Der p 7 by Inhibition of IgE Binding With Peptide-Specific Antisera

The fact that Der p 7-sensitized allergic patients showed no IgE reactivity to Der p 7-derived peptides suggested that IgE epitopes of Der p 7 are likely discontinuous and/or conformational epitopes similar as has been observed for many other respiratory allergens (34). It is possible to map such epitopes with the use of peptide-specific antibodies that can recognize the corresponding folded complete antigen by competitive binding experiments (35). The principle of this indirect mapping of IgE epitopes is that peptides-specific antibodies upon binding to the folded allergen block the corresponding peptide indicating that IgE antibodies bind to the region where the peptide is located (32, 33). We pre-incubated Der p 7 with Der p 7-peptide-specific antisera and studied if the rabbit antibodies can inhibit allergic patients IgE binding to Der p 7. **Table 1** shows the percentages of inhibition of patients’ IgE binding to Der p 7 obtained by pre-incubation of the allergen with the individual peptide-specific antibodies and with antibodies raised against the complete folded allergen for ten Der p 7-sensitized HDM allergic patients. The ten HDM allergic patients suffered from HDM-related respiratory symptoms (rhinitis and/or asthma). Antisera were obtained with two different adjuvants, aluminium hydroxide and Freund’s adjuvant (CFA) (**Table 1**). The extent of inhibition of IgE binding was stronger with antisera obtained with CFA as adjuvant as compared to antisera obtained with aluminium hydroxide-adjuvanted antigens (**Table 1**) which may be due to the fact that CFA-adjuvanted antigens induced higher Der p 7-specific IgG levels than aluminium hydroxide adjuvanted antigens (**Figure S3**). However, the qualitative results in terms of peptides involved in IgE binding were comparable. The strongest inhibition of IgE binding was achieved with anti-peptide 1 antisera (**Table 1**). When Alum was used as an adjuvant for immunization of the rabbits, the highest mean inhibition was obtained by anti-P1 antibodies (i.e., 66% mean inhibition) which was even higher than with anti-Der p 7 antibodies (i.e., 57% mean inhibition) (**Table 1**, upper panel). Somewhat lower inhibitions were obtained by anti-P2, anti-P3, and anti-P7 antibodies (i.e., mean inhibitions of 26%, 27% and 22%, respectively). Inhibitions lower that 20% were obtained with anti-P4 (i.e., 16% mean inhibition) and no relevant inhibition could be obtained with anti-P5 and anti-P6 antibodies (**Table 1** **)**. When CFA was used as adjuvant, the mean inhibition obtained with anti-Der p 7 was 83%, the highest inhibitions with anti-peptide antisera were obtained with anti-P1 and anti-P2 antibodies (mean inhibitions of 65% and 62%, respectively) followed by anti-P7, anti-P4, and anti-P3 (mean inhibitions of 52%, 46% and 33%, respectively (**Table 1**, lower panel). Like for aluminium hydroxide-adjuvanted antisera, no relevant inhibition was found for anti-P6 and anti-P5 antibodies. These results indicate that IgE epitopes are present on two sites of the Der p 7 molecule, one area defined by peptides 2 and 7 at the “front side” of Der p 7 (**Figure 2**) and another area defined by peptides 1, 3, and 4 on the “back side” of Der p 7 (**Figure 2**). These two epitope-containing areas seem to belong to the discontinuous type of epitopes because peptides 2 and 7 as well as peptides 1 and 3 to 4 are not adjacent in the Der p 7 sequence. A consistent inhibition of IgE binding was observed with anti-P1 antibodies which was almost as high as with anti-Der p 7 antibodies regardless of the levels of Der p 7-specific IgE antibodies (**Table 1**).

**Table 1:** Inhibition of patients` IgE binding to Der p 7 by sera against Der p 7 peptides.

Alum										
% inhibition										
patient	anti-Der p 7	anti-P1	anti-P2	anti-P3	anti-P4	anti-P5	anti-P6	anti-P7		
19	62	81	23	43	5	3	0	48		
20	65	62	6	16	11	0	5	9		
21	69	78	11	38	14	0	2	29		
22	63	75	30	30	9	8	0	23		
24	78	75	36	49	22	0	15	42		
25	41	68	40	36	36	3	23	18		
26	46	60	32	7	7	0	3	13		
27	35	30	13	0	0	0	0	2	**patient**	**OD**
28	32	58	32	26	45	1	24	26	19	0.721
29	75	72	36	29	12	1	3	10	20	0.999
**Mean**	**57**	**66**	**26**	**27**	**16**	**2**	**8**	**22**	21	0.848
									22	0.818
**CFA**	** **	** **	** **	** **					24	0.368
**% inhibition**									25	0.282
**patient**	**anti-Der p 7**	**anti-P1**	**anti-P2**	**anti-P3**	**anti-P4**	**anti-P5**	**anti-P6**	**anti-P7**	26	0.426
19	91	79	75	43	72	2	4	67	27	0.660
20	84	68	60	31	33	3	8	37	28	0.341
21	93	76	70	38	61	0	15	62	29	1.809
22	90	67	68	25	49	0	8	52		
24	75	76	73	58	30	0	18	59		
25	85	54	60	23	41	1	5	50		
26	81	52	64	34	47	6	11	60		
27	80	36	38	25	42	0	2	29		
28	67	61	48	33	53	10	3	49		
29	96	79	62	21	35	0	20	57		
**Mean**	**83**	**65**	**62**	**33**	**46**	**2**	**9**	**52**		

The percentages of inhibition of patients’ (19-22,24-29) IgE binding to Der p 7 after preincubation with the antisera derived against Der p 7 peptides (P1-P7) or with antiserum derived against Der p 7. Der p 7-specific IgE levels as determined by ELISA and expressed as OD values were indicated in the last column.

## Discussion

House dust mite is one of the most important respiratory allergen sources worldwide, and there is a need for effective vaccines for AIT of HDM allergy. Two recent studies provided evidence that HDM allergen extract-based AIT vaccines provide mainly protection against Der p 1 and Der p 2 whereas they induce low or no IgG antibody responses to Der p 5, Der p 7, Der p 21, and Der p 23, and treatment success is lower in patients who are sensitized against the latter allergens as compared to patients who are mainly sensitized to Der p 1 and/or Der p 2 (17, 18). Der p 7 has been defined as a mid-tier house dust mite allergen recognized by more than 30% of HDM sensitized patients (3) but it has been shown that it can be a major HDM allergen recognized by more than 50% of HDM allergic patients in certain populations (5, 9). However, not only the frequency of IgE recognition defines if an allergen is important but also its clinical relevance which may be measured by determining its allergenic activity *in vitro* and *in vivo* needs to be taken into consideration as has been suggested recently (36, 37). We expressed in *Escherichia coli* and purified recombinant Der p 7 and demonstrated that the molecule is folded by CD spectroscopy. rDer p 7 induced dose-dependent basophil activation starting already at low concentrations in the nanogram per milliliter range confirming earlier results obtained by skin prick testing (10) which suggested high allergenic activity of Der p 7. Furthermore, our study confirmed that IgE recognition of Der p 7 is associated with respiratory symptoms as has been observed earlier (11). It is a limitation of our study that the cohort of patients used to investigate possible associations of IgE reactivity to Der p 7 with clinical symptoms was an AIT population in which severe forms of asthma were excluded by definition. However, the use of HDM-allergic patients eligible for AIT was also a strength, because it showed that a substantial portion of the AIT patients (i.e., 44%) was sensitized to Der p 7, confirming that Der p 7 is an essential component to be included in a HDM AIT vaccine.

A major limitation of currently used HDM allergen extract-based vaccines is that natural allergen extracts lack important allergens such as Der p 5, Der p 7, Der p 21, and Der p 23 as immunogenic component (16–18) and therefore allergen extract-based vaccines seem to be less effective in patients sensitized to these allergens (17, 18).

Accordingly it has been proposed to develop molecular AIT vaccines which can be designed to include all relevant allergens of a given allergen source as has been demonstrated for the grass pollen allergy vaccine BM32 which includes the four major timothy grass pollen allergens Phl p 1, Phl p 2, Phl p 5, and Phl p 6 in the form of non-allergenic fusion proteins (38). A major step in the design of B cell epitope-based recombinant hypoallergenic vaccines like BM32 is the definition of IgE epitopes of the allergens to be included and the identification of non-allergenic peptides derived from the IgE epitopes so that they can be fused with an immunological carrier. The fusion protein consisting of the non-allergenic IgE epitope-derived peptides of the allergen and an unrelated carrier (i.e., hepatitis B-derived preS in case of the BM vaccines) can then be used to induce IgG antibodies toward the IgE epitopes of the allergen which block allergic reactions by interfering with the IgE recognition of the allergen.

Our study can be considered as an important step toward the creation of a HDM AIT vaccine based on carrier-bound non-allergenic IgE epitope-derived peptides, because we succeeded to define IgE epitopes of Der p 7 and to identify non-allergenic peptides derived from the IgE binding sites of Der p 7 which upon coupling to a carrier induced IgG antibodies blocking allergic patients IgE binding to Der p 7.

To achieve this goal we used sera from HDM allergic patients to study the nature and localization of the IgE epitopes on Der p 7. For this purpose we used two different approaches: First we synthesized seven peptides of 27 to 37 amino acids length which according to structural prediction were surface-exposed to search for the presence of sequential epitopes but none of the tested allergic patients showed IgE reactivity to the peptides. Thus, Der p 7, similar as most of the potent respiratory allergens (25, 39), seems to lack relevant sequential IgE epitopes. A study by Chou et al. (40) showed by dot blot inhibition experiments that synthetic peptide (15-mer) containing the sequence (aa156–160: SILDP) showed IgE reactivity in two out of 30 asthmatic serum samples. The study suggested that asparagine at position 159 might be crucial for IgE cross-reactivity between Der p 7 and Der f 7 but the asparagine was identified by mutation experiments which may affect the structure of the complete allergen, its surface and/or directly its IgE interaction. Although the amino acid sequence SILDP is identical between Der f 7 and Der p 7 we did not find IgE binding of the patient’s sera with peptide 6 which contains this sequence. In order to determine areas on the Der p 7 structures which are part of the conformational epitopes we then followed an indirect strategy for IgE epitope mapping which has been suggested for antigens earlier (35) and has already allowed to map IgE epitopes of several important respiratory allergens (25, 32, 33, 39, 41, 42). We raised rabbit antisera against the allergen-derived peptides and then used these rabbit antisera to block the binding of allergic patients IgE to the complete folded Der p 7 allergen. Using the indirect IgE epitope mapping approach we identified two IgE-reactive areas, one on the front (area 1) and one on the back (area 2) side of the Der p 7 molecule which are assembled by non-adjacent peptides (area 1: peptides 1, 3, and 4; area 2: peptides 2 and 7). Our results thus indicate that Der p 7 contains two conformational, discontinuous IgE-epitope containing areas. It was interesting that some anti-Der p 7-peptide sera obtained with CFA as adjuvant showed different IgE inhibitory capacity (anti-P2, anti-P4, anti-P7, anti-Der p 7) to antisera raised against the same peptides with Alum as adjuvant, whereas for other peptides no such differences were observed (anti-P1, anti-P3, anti-P5, anti-P6). Since CFA is known to be a more potent adjuvant in rabbits than Alum the higher inhibitions with antisera raised with CFA (i.e., anti-P2, anti-P4, anti-P7) may be explained by the high antibody titers. This was also observed for anti-Der p 7 antisera. In the case of anti-P1 and anti-P3, higher titers were achieved with Alum than with CFA or in the case of anti-P5 and anti-P6 similar titers were achieved with Alum and CFA and inhibition levels were comparable for CFA- and Alum-adjuvanted antisera.

Interestingly, antibodies directed toward the N-terminal peptide 1 were almost as potent as antibodies raised against the complete folded Der p 7 allergen to block IgE binding. Since the goal of this study was to identify peptides to be included in vaccines for treatment of HDM allergic patients we selected P1 because it induced a strong immune response and good inhibition capacity almost comparable to Der p 7 when Alum, an adjuvant allowed for human use was employed. We therefore suggest to consider peptide 1 together with peptides with similar properties derived from Der p 1, 2, 5, 21, and 23 into a peptide carrier-based vaccine for treatment of HDM allergy.

Besides having defined IgE epitopes of Der p 7 our work thus makes a contribution to the future development of a hypoallergenic HDM allergy vaccine based on the peptide-carrier fusion technology.

## Data Availability Statement

The original contributions presented in the study are included in the article/**Supplementary Material**. Further inquiries can be directed to the corresponding author.

## Ethics Statement

The studies involving human participants were reviewed and approved by the ethics committee of the Medical University of Vienna, Austria (EK 641/2014). The patients/participants provided their written informed consent to participate in this study.

## Author Contributions

MC and RV designed the study, interpreted the results, and wrote the manuscript. MC, H-JH, TG, SG, K-WC, YR-M, and KF conducted experiments, analyzed the data, and prepared the figures. WH, PZ, RZ, PL, and FH collected allergic patients’ sera and performed clinical characterization of patients. MF-T, SV, SF, and WK analyzed and interpreted the results. RV supervised and coordinated the study. All authors contributed to the article and approved the submitted version.

## Funding

This study was supported by grants F4602, F4604, F4605, and F4607 of the Austrian Science Fund (FWF), by the Danube Allergy Research Cluster, Country of Lower Austria, and by a research grant from WORG Pharmaceuticals, Hangzhou, China. The funder, WORG Pharmaceuticals, was not involved in the study design, collection, analysis, interpretation of data, the writing of this article or the decision to submit it for publication.

## Conflict of Interest

RV has received research grants from Viravaxx, Vienna, Austria, HVD Biotech, Vienna, Austria and WORG Pharmaceuticals, Hangzhou, China and serves as consultant for Viravaxx and WORG.

The remaining authors declare that the research was conducted in the absence of any commercial or financial relationships that could be construed as a potential conflict of interest.

## Supplementary Material

Supplementary Figure 1 Surface accessibility of amino acids calculated for the Der p 7 peptides according to the three-dimensional structure of Der p 7. Shown are the average relative surface exposures of amino acids of the Der p 7-derived peptides (x-axes) compared to the G-X-G tripeptide accessibility of each respective amino acid (y-axes). The Der p 7-derived peptides are indicated below the plot in different colors.

Supplementary Figure 2 Characterization of purified recombinant Der p 7**(A)** Coomassie brilliant blue-stained SDS-PAGE containing purified recombinant Der p 7 separated under reducing conditions. Molecular weight markers are indicated on the left margins in kilo Dalton (kDa). **(B)** Far-UV circular dichroism (CD) analysis of Der p 7. Shown are mean residue ellipticities (mre) at the given wavelengths (x-axis). **(C)** Mean residue ellipticities (mre) determined for rDer p 7 (y-axis) at a given wavelength at different temperatures by CD (x-axis).

Supplementary Figure 3 Immunization with Der p 7-derived peptides induces Der p 7-specific antibodies in rabbits. Rabbits were immunized with KLH-coupled Der p 7-derived peptides adsorbed to alum (2 rabbits per peptide) or to CFA (2 rabbits per peptide). IgG reactivity of sera from immunized rabbits to ELISA plate-bound Der p 7 was determined for different dilutions (x-axes) of the anti-sera (anti-Der p 7 P1; anti-Der p 7 P2; anti-Der p 7 P3; anti-Der p 7 P4; anti-Der p 7 P5; anti-Der p 7 P6; anti-Der p 7 P7. Anti-Der p 7 antisera and corresponding pre-immune sera (PI) were included for comparison. Data are shown as optical density (OD) values (y-axes).

## Abbreviations

ELISA, enzyme-linked immunosorbent assay; AIT, allergen-specific immunotherapy; OD, optical density; KLH, Keyhole Limpet Hemocyanin.
