# Supplementary figures and images for: IgE Epitopes of the House Dust Mite Allergen Der p 7 Are Mainly Discontinuous and Conformational
Source: Front Immunol. 2021 Jun 15;12:687294. doi: 10.3389/fimmu.2021.687294 (PMC8241568; doi:10.3389/fimmu.2021.687294)

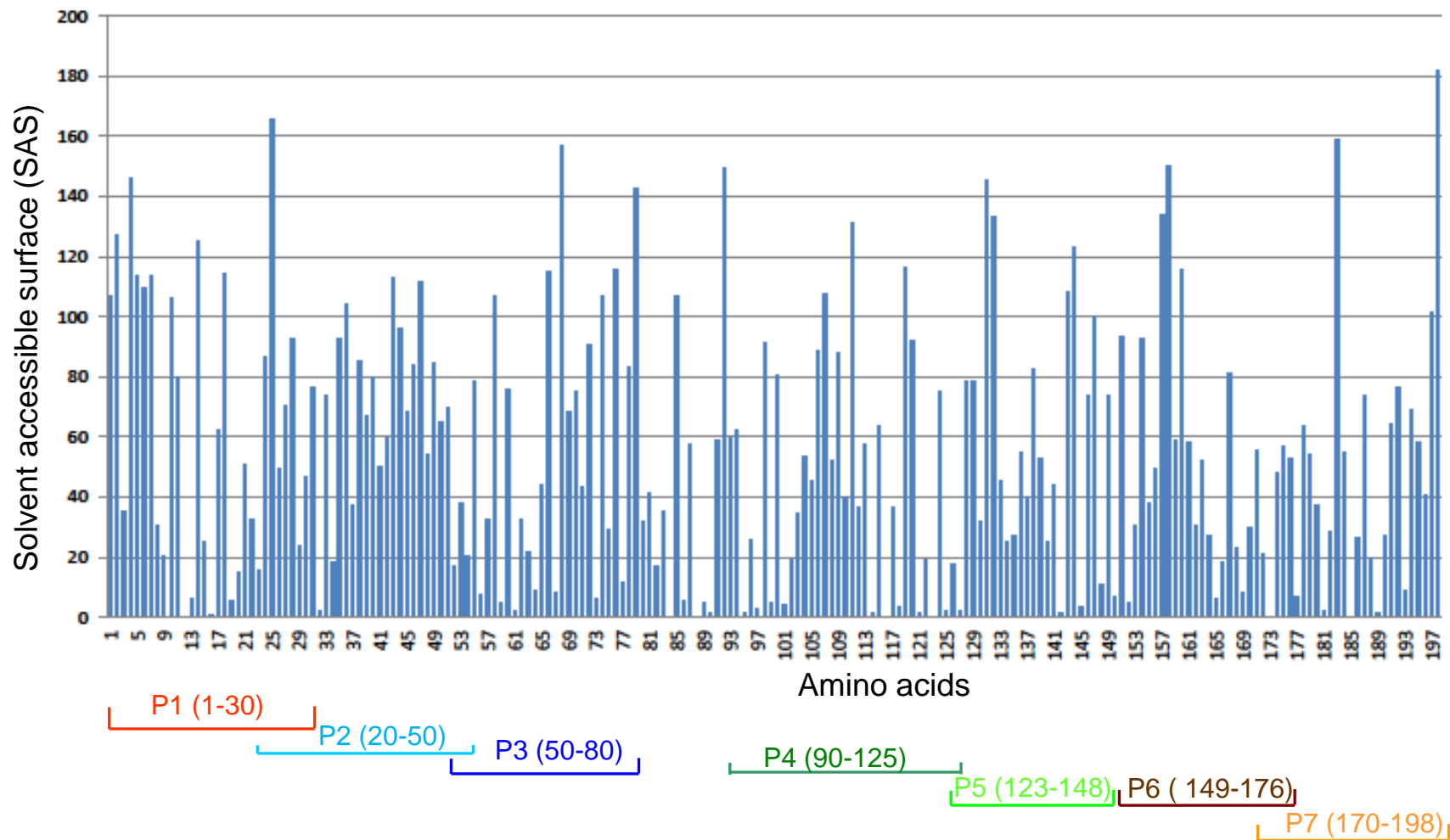

Figure S1

Supplement: Supplementary Figure 1 — Surface accessibility of amino acids calculated for the Der p 7 peptides according to the three-dimensional structure of Der p 7. Shown are the average relative surface exposures of amino acids of the Der p 7-derived peptides (x-axes) compared to the G-X-G tripeptide accessibility of each respective amino acid (y-axes). The Der p 7-derived peptides are indicated below the plot in different colors. [file DataSheet_1.pdf]
