# Supplementary material for: IgE Epitopes of the House Dust Mite Allergen Der p 7 Are Mainly Discontinuous and Conformational
Source: Front Immunol. 2021 Jun 15;12:687294. doi: 10.3389/fimmu.2021.687294 (PMC8241568; doi:10.3389/fimmu.2021.687294)
Supplement: Supplementary file 4 [file DataSheet_2.pdf]

Table S1. Characteristics of synthetic Der p 7-derived peptides

Peptide	Position (aa)	Sequence	No. of amino acids	Molecular mass (Da)	Isoelectric point
peptide 1	1-30	**C**DPIHYDKITEEINKAVDEAVAAIEKSETFD	31	3494,83	4,20
peptide 2	20-50	**C**VAAIEKSETFDPMKVPDHSDKFERHIGIIDL	32	3642,16	4,96
peptide 3	50-80	**C**LKGELDMRNIQVRGLKQMKRVGDANVKSEDG	32	3589,16	9,11
peptide 4	90-125	**C**VHDDVVSMEYDLAYKLGDLHPNTHVISDIQDFVVEL	37	4230,73	4,18
peptide 5	123-148	**C**VELSLEVSEEGNMTLTSFEVRQFANV	27	3032,39	3,98
peptide 6	149-176	**C**VNHIGGLSILDPIFAVLSDVLTAIFQDT	29	3072,57	3,93
peptide 7	170-198	**C**TAIFQDTVRAEMTKVLAPAFKKELERNNQ	30	3452,99	8,18

Cysteines added for coupling to KLH are marked in bold
